# Longitudinal team training program in a Norwegian surgical ward: a qualitative study of nurses’ and physicians’ experiences with implementation

**DOI:** 10.1186/s12913-021-06732-6

**Published:** 2021-07-23

**Authors:** Randi Ballangrud, Karina Aase, Anne Vifladt

**Affiliations:** 1 Department of Health Sciences Gjøvik, Faculty of Medicine and Health Sciences, Norwegian University of Science and Technology, Teknologivn. 22, 2815 Gjøvik, Norway; 2 Center for Resilience in Healthcare, Faculty of Health Sciences, University of Stavanger, Kjell Arholms hus, Kjell Arholms gate 43, 4021 Stavanger, Norway

**Keywords:** Interprofessional teamwork, Patient safety, Team training program, Qualitative research

## Abstract

**Background:**

Team training interventions to improve team effectiveness within healthcare are widely used. However, in-depth knowledge of how healthcare professionals experience such team training curricula and their implementation processes, as well as how contextual factors impact implementation, is currently missing. The aim of this study is therefore to describe healthcare professionals’ experiences with the implementation of a longitudinal interprofessional team training program in a surgical ward.

**Methods:**

A descriptive design was applied based on qualitative semi-structured focus group interviews with 11 healthcare professionals. A convenience sample of physicians (*n* = 4), registered nurses (*n* = 4), and certified nursing assistants (*n* = 3) was divided into three professionally based focus groups, which were interviewed at three time intervals over a period of 1 year.

**Intervention:**

The validated and evidence-based team training program Team Strategies and Tools to Enhance Performance and Patient Safety (TeamSTEPPS) was implemented in a surgical ward at a hospital between January 2016 and June 2017. The team training program included three phases: 1) assessment and planning, 2) training and implementation, and 3) sustainment.

**Results:**

Healthcare professionals’ experiences with the content of the team training program varied from valuing the different elements of it to seeing the challenges in implementing the elements in clinical practice. A one-day training course was found to be especially beneficial for interprofessional collaboration at the ward. Over time, the nursing staff seemed to maintain their motivation for the implementation of the tools and strategies, while the physicians became less actively involved. Contextual ward factors influenced the adoption and utilization of the tools and strategies of the program both positively and negatively. The healthcare professionals’ experienced the implementation of the team training program as positive for the patient safety culture at the ward in the forms of increased awareness of teamwork and open communication.

**Conclusions:**

The study suggests that the implementation of a team training program in a surgical ward is dependent on a set of factors related to content, process, context, and impact. Knowledge on how and why a team training program work supports the transferability to clinical practice in further planning of team training measures.

**Trial registration:**

The study is part of a larger research project with a study protocol that was registered retrospectively on 05.30.17, with the trial registration number ISRCTN13997367.

**Supplementary Information:**

The online version contains supplementary material available at 10.1186/s12913-021-06732-6.

## Background

Interventions to improve team effectiveness within healthcare are widely used [1]. Team training is one such intervention that has been described by Hughes et al. [2] as a learning strategy in which a group systematically acquires teamwork-related knowledge, skills, and abilities that impact the cognitions and behaviors of a team. A plethora of team training interventions exist, yet most studies have examined the use and evaluation of self-reported surveys, external assessments of behavior, and satisfaction surveys [3]. Exploring how healthcare professionals experience team training curricula and implementation processes that apply qualitative methods is mainly missing. There is also a need for more research on how contextual factors impact team effectiveness. Cunningham et al. [4] identified the following five contextual enablers for teams to be effective: (a) interprofessional focus and a flattened hierarchy, (b) effective communication, (c) leader support and the alignment of team goals with the organizational goals, (d) the credibility of the interventions, and (e) suitable team structure with physician involvement.

The current study reports the implementation process of a longitudinal interprofessional team training intervention in a surgical ward based on the Team Strategies and Tools to Enhance Performance and Patient Safety (TeamSTEPPS) program. TeamSTEPPS is an acknowledged, validated, and evidence-based team training program [5] that has been used in various healthcare settings and countries [3]. The TeamSTEPPS curriculum focuses on five key principles, namely, team structure communication, leadership, situation monitoring, and mutual support, and it provides tools and strategies that can be used to achieve competency in each area. The program’s basic assumption is that these five teamwork principles are critical for safe patient care [5].

TeamSTEPPS is designed to improve the quality, safety, and efficiency of health care. A key element in implementing the program is to strengthen the culture of patient safety [5]. Patient safety culture is complex and consists of several subcultures, such as leadership, teamwork, and communication [6]. The introduction of interventions such as team training and communication tools is recommended to promote patient safety culture [7], which is similar to the tools and strategies included in the TeamSTEPPS training program (AHRQ, 2012).

A systematic review by Welsch et al. [8], found that interprofessional team training incorporating a TeamSTEPPS didactic session and high-fidelity simulation results in positive changes in a variety of outcome measures, such as satisfaction with the team training program, teamwork attitude, perceptions of teamwork, team performance, efficiency in inpatient care, and patient outcome. Other studies investigating the program’s implementation in a number of hospitals have found positive changes in patient safety culture [9, 10].

A key question in studying the implementation of team training interventions is whether they are found to be effective or useful in everyday clinical practice by the healthcare personnel who are the main target group for these interventions [11]. Questions such as whether the team training program induces the expected changes at a hospital ward or unit, or whether the implementation process is achievable represent the issues that are vital to gaining knowledge. To study healthcare professionals’ experiences with these issues, we applied the Pettigrew, Whipp [12] model for strategic change, which consists of the three dimensions of content, process, and context. In addition, the dimension of impact was added to better understand the resulting changes present in the team training program. The model of strategic change framed the study in terms of producing in-depth qualitative knowledge of the elements and components of the team training program (content); the methods, strategies, and implementation measures taken (process); the local issues influencing the implementation (context); and the resulting changes in the patient safety culture of clinical practice (impact).

To our knowledge, this is the first qualitative study in a Norwegian setting that explores how healthcare professionals experience the implementation of the TeamSTEPPS program in a hospital ward. Therefore, the main aim of the study is to describe healthcare professionals’ experiences with the implementation of a longitudinal interprofessional team training program in a surgical ward. The following research question guided the study: How do nurses and physicians in a surgical ward experience the contents of the team training program, the process of implementing the program, the context in which the program is implemented, and the impact of the program on the ward’s patient safety culture? The study is part of a larger research project on teamwork in hospitals [13], and it is the second report on qualitative data from this project; the first studied healthcare professionals’ experiences with teamwork skills [14].

## Methods

### Design

The study used a descriptive design based on qualitative semi-structured focus group interviews with healthcare professionals at three time intervals.

### Setting

The implementation of the team training program was carried out in a surgical ward (20-bed combined gastrointestinal surgery and urology) of a hospital division (200 beds) in a Norwegian hospital trust from May 2016 and June 2017. The surgical ward was selected as the intervention ward due to its motivation for quality improvement and patient safety after recently having experienced several adverse events. The management group consisted of two nurses (the unit nurse manager and the clinical nurse specialist) and two physicians (the head surgeon of urology and the head surgeon of gastrointestinal surgery). The head of the department supported the intervention. The healthcare professionals were organized into two interprofessional teams carrying out patient care. The ward’s bed occupancy rates, patient admissions, and number of full-time equivalent positions are reported in Table 1, before and during implementing the team training program.

**Table 1:** Ward information data before and during the team training program

	Before	Six months	12 months
**Bed occupancy and patient admissions**^a^			
Number of beds	20	20	20
Occupied beds	87%	96%	89%
Admissions per month	192	174	173
Length of stay (mean days)	3.46	3.63	3.62
Emergency admissions	64%	65%	66%
**Healthcare professionals** (full-time equivalent positions)			
Physicians	13	12	12
Registered nurses (RN)	17.25	19.25	20.25
Certified nursing assistants (CNA)	4.95	3.1	2.1
Unit nurse manager	1.0	1.0	1.0
Clinical nurse specialist	1.0	1.0	1.0
**Changes in number of positions**			
Clinical nurse specialist	–	No	No
Unit nurse manager	–	No	Yes
Head physician gastrointestinal surgery	–	No	Yes
Head physician urology	–	No	Yes
Chair of the surgical department	–	No	Yes

^a^Based on prior six-month period

### Sample

A convenience sample of 11 healthcare professionals (informants) recruited from the surgical ward was divided into three focus groups based on RNs (*n* = 4), CNAs (*n* = 3), and physicians (*n* = 4). The inclusion criterion was that the healthcare professionals had to have participated in a one-day TeamSTEPPS training course. A total of 41 healthcare professionals participated in the mandatory training. Information about the study and the research team was distributed to all the healthcare professionals, 11 of whom confirmed their participation in the study. The sample consisted of eight women and three men with varying work experiences and employment type within the surgical ward. To ensure the informants’ anonymity, no further background information is provided in the paper.

### Intervention

The intervention consisting of the TeamSTEPPS training program was initiated by a research team as part of a larger research project [13]. The TeamSTEPPS program and the related teaching materials were translated into Norwegian by the research team. The management group in the surgical ward had the main responsibility for the training and implementation of the program. Before implementation, the management group attended a TeamSTEPPS 2.0 Master Training Course in the USA and were thus certified as instructors. The team training program was planned and implemented according to the TeamSTEPPS implementation plan in three phases [5], built on John Kotter’s model with eight steps for organizational change [15]. The intervention was conducted in the period from January 2016 to June 2017.

#### Phase 1) setting the stage and deciding what to do—assessment and planning

To create an awareness of the need for a team training program, short information meeting and a lesson about teamwork in relation to patient safety were organized by the research group for the physicians and nursing staff. The management group decided that the ward was ready for the TeamSTEPPS intervention after reviewing the collected assessment profile of the surgical ward. A training and implementation plan was established.

#### Phase 2) making it happen—training and implementation

A one-day interprofessional team training course in an external simulation center was conducted for all healthcare professionals at the ward during 3 weeks in May 2016. The training comprised 6 hours of classroom training (lectures, videos, role plays and discussions) and 2 hours of high-fidelity simulation, focusing on patient safety and the TeamSTEPPS five key principles. At the end of the course, all healthcare professionals were asked to point out relevant patient safety issues in their clinical practice, and to recommend TeamSTEPPS tools to solve them. After the team training course, a Change Team with a guiding role was assigned and led by the Nurse Unit Manager. The Change Team had members from all ward professions (two RNs, two CNAs and four physicians), in addition to the Chair of the surgical department, a former patient, and a researcher serving as a coach. An action plan for the implementation was established, based on the identified patient safety issues at the ward and communicated at unit staff meetings and via email to all employees. Five TeamSTEPPS tools were implemented during the first 6 months of the team training period, launched as “The tool of the month” (Table 3) and communicated in the weekly newsletters sent to the healthcare professionals. Short-term wins were celebrated at the ward, e.g. after the implementation of the ISBAR tool (the Norwegian word for ice cream bar) healthcare professionals were invited to a provisional ice cream bar at the ward. Five months after the initial one-day team training course a TeamSTEPPS refresher course was organized with a duration of 75 min for the nursing staff and 20 min for the physicians.

#### Phase 3) making it stick—sustainment (January 2017–June 2017)

The implementation in line with the action plan continued, with five more tools implemented. Another TeamSTEPPS refresher course wase held for the nursing staff.

An overview of the implementation of the team training program with belonging phases, timeline, and activities is presented in Table 2. A more detailed description of the team training program can be found in Aaberg et al. 2019 [16].

**Table 2:** The TeamSTEPPS intervention

Phase 1) Setting the stage and deciding what to do—assessment and planning	Phase 2) Making it happen—training and implementation	Phase 3) Making it stick—sustainment
January 2016–April 2016	May 2016–December 2016	January 2017–June 2017
▪ Site assessment. ▪ Short information meetings and a lesson about teamwork and patient safety. ▪ The management decided the ward ready for TeamSTEPPS ▪ A training and implementation plan established.	▪ One-day interprofessional team training course. ▪ All healthcare professionals pointed out patient safety issues and recommend TeamSTEPPS tools to solve them. ▪ A change team established. ▪ An action plan established. ▪ Five TeamSTEPPS tools implemented. ▪ Short-term wins celebrated. ▪ TeamSTEPPS refresher training course.	▪ Implementation continued. ▪ Five new TeamSTEPPS tools implemented. ▪ All initiatives coached, monitored and integrated. ▪ Short-term wins celebrated. ▪ Additional TeamSTEPPS refresher courses.

Table 3 displays the TeamSTEPPS tools and strategies implemented during the 12 month training and implementation period.

**Table 3:** Implementation of TeamSTEPPS tools and strategies

Time		Tools and strategies	Implementation arena
**2016**	May	Closed loop	Exchange of critical information
	Jun	ISBAR^a^	Communicating of critical patient information
	Aug	Briefs	Start of every shift
	Sept	Huddles	Redistribution of tasks at patient safety whiteboard meetings
	Oct	Cross-monitoring	Double control by intravenous medication administration
**2017**	Jan	Debriefs	Manager with nursing staff, once a week
		Task Assistance	Distribution of workload
	Feb	STEP^b^	Updated in electronic care plan
	Mar	Two-Challenge Rule	When an initial assertive statement is ignored
	May	I-PASS^c^	Handoffs with focus on patient safety risks

^a^*ISBAR* **I**ntroduction, **S**ituation, **B**ackground, **A**ssessment, **R**ecommendation

^b^*STEP* **S**tatus of the patient, **T**eam members, **E**nvironment, **P**rogress towards the goal

^c^*I-PASS* **I**llness severity, **P**atient summary, **A**ction list, **S**ituation awareness and contingency planning, **S**ynthesis by receiver

### Data collection

The data collection was based on focus group interviews with healthcare professionals conducted before and after six and 12 months of the implementation of the intervention. A total of ten focus group interviews with healthcare professionals (informants) were conducted. Three initial focus group interviews were completed with each professional group before the start of the intervention. Four follow-up interviews were conducted after 6 months of the team training program, with three consecutive follow-up interviews being conducted after 12 months. See Table 4 for more information.

**Table 4:** Focus group interviews held before and six and 12 months after the intervention

Before intervention^**a**^	After 6 months	After 12 months
Interviews (*n* = 3) *April 2016*	Interviews follow up (*n* = 4) *November 2016*	Interviews follow up (*n* = 3) *June 2017*
RNs (*n* = 4)	RNs (*n* = 3)	R.N.s (*n* = 3)
CNAs (*n* = 2)	CNAs (*n* = 2)	CNAs (*n* = 2)
Physicians (*n* = 3)	Physicians (*n* = 2)	Physicians (*n* = 1)
	Physicians (*n* = 2)	

^a^The TeamSTEPPS intervention started on May 2016

To validate the thematic interview guides, a pilot interview was conducted which generated a new question (experiences related to the TeamSTEPPS refresher courses), resulting in the final version of the guides. Two researchers (RB, AV) conducted the focus group interviews, with one researcher as a moderator and the other as an observer taking notes. All the interviews were conducted in a meeting room at the hospital during the daytime. The interviews started with a clarification of the study’s aims and were conducted as a dialogue in which the informants were encouraged to complete an open-ended collective activity with a reflection on common experiences [17]. The focus groups were asked the same questions, and follow-up questions were used to encourage the informants to elaborate on and/or clarify their responses [18]. AV served as a moderator at the initial interviews conducted before the intervention, while RB served as a moderator at the interviews at six and 12 months. The initial interviews before the start of the intervention referred to the different healthcare professionals’ expectations about the team training program and their perceptions of the teamwork climate in the surgical ward. At six and 12 months, the interview questions referred to the healthcare professionals’ experiences with the implementation of a team training program in the surgical ward (see Additional file 1). The participants approved a summary of the interview notes directly after each interview, which lasted from 25 to 60 min each (mea*n* = 33 min). All the interviews were digitally recorded, transcribed verbatim and anonymized before the analysis.

### Data analysis

The interviews were analyzed by using a deductive manifest content analysis approach according to Elo, Kyngäs [19]. The framework used in the deductive analysis was based on the Pettigrew, Whipp [12] model, which distinguishes between the three dimensions of strategic change, namely, content, process and context. 1) The *content* of the team training program refers to what the informants mean about the specific content of the TeamSTEPPS program, including teamwork skills, tools and strategies. 2) The *process* of implementing the program refers to how the informants experienced the one-day team training course and the implementation of the program in the surgical ward. 3) The *context* in which the program was to be implemented refers to where the data were collected and is concerned with the cultural, environmental, or other internal and/or external factors in the surgical ward that influenced the implementation of the intervention. Additionally, 4) the *impact* of the team training program on achieving a patient safety culture at the ward refers to how the informants experienced the influence of the team training program on patient safety culture in their daily practices.

According to Elo, Kyngäs [19], the preparation phase, the organizing phase and the reporting phase are the basis of an analysis. The entire research team (RB, KA and RB) contributed to the data analysis. In the preparation phase the transcripts of the focus group interviews were handled manually and systematized into a Word table without using any software tool. Each interview was defined as one unit of analysis, and the data from before, after 6 months and after 12 months of the intervention were analyzed separately. To become familiar with the data and guided by the study aim and the research question, all the interviews were read several times by all three authors (RB, KA and AV). In the *organization phase,* a structured analysis matrix with columns representing the categories of content, context, process and impact was established. All the data were reviewed for content and were coded according to the four categories first individually by RB and AV. To ensure trustworthiness in the analysis [20], the two preliminary individual analyses were discussed face-to-face in a meeting among the three authors (RB, KA and AV) until agreement was reached. Table 5 displays an example from the codebook.

**Table 5:** Example from codebook related to the four categories of content, process, context, and impact

Content	Process	Context	Impact
RN.2.17 The tools are related to strategies used previously but they have not been named before.	Ph.2.7. It was useful to spend time together with the nurses during a course day.	CNA.3.22 If it gets busy, then the whiteboard meeting is not prioritized.	RN.2.21 By using the tools, issues are detected that have not previously been detected.
Ph.2.20 The principles related to systematics are good.	RN:2.14 It was very useful to have the information repeated at the refresher course.	Ph.3.44 There is much knowledge present within the ward.	CNA.2.55. We have become more aware of commenting on each other.
CNA.3.33. Debriefing can form the basis for change.	CNA.3.57 To get the knowledge to last, one should have it incorporated so that it becomes routine.	RN.2.134 Time is a critical factor in using the program.	Ph.3.12 More members may have become aware of their communication using phones, ward rounds, and verbal prescriptions.

The matrix revealed 501 condensed units of meaning representing the four categories. In the *reporting phase*, the results were presented based on a summarized content of each of the four categories, thereby representing the information provided by the informants. Quotations were used to enhance and illuminate the categories [21]. Continuous discussion among all three authors was prominent throughout the reporting phase to secure trustworthiness [20]. The results were reported according to the Consolidated Criteria for Reporting Qualitative Research (COREQ) [22].

## Results

The results are presented with the aim of describing healthcare professionals’ experiences with the implementation of a team training program in a surgical ward according to the following four categories: (a) *the content of the team training program*, (b) *the process of implementing the program*, (c) *the context in which the program is to be implemented,* and (d) *the impact of the program on patient safety culture in the ward.*

### The content of the team training program

The healthcare professionals reported many different experiences related to the content of the team training program, ranging from those that valued the different tools and strategies of the program to those that saw them as challenging and difficult to incorporate in daily clinical practice in the ward. The experiences differed within and between professional groups and were often formed by the expectations that the participants had about the team training program.

Prior to the implementation of the team training program, registered nurses (RNs) and certified nursing assistants (CNAs) expressed positive attitudes towards and had open minds about taking part in the training activities, while physicians expressed more skeptical attitudes that were rooted in their view of the already satisfactorily teamwork present in the ward.

During the program period (six and 12 months after implementation), RNs continued to experience the different components of the program positively. They already knew several of the components from beforehand; however, with designated names given to team training strategies and tools, their care practices were systematized, and their awareness of current team practices increased. During the program period, physicians and CNAs reported that a plethora of the labels on the strategies and tools were confusing, and the CNAs missed the Norwegian terms for the tools. While the CNAs adjusted to the labels over time, the physicians continued to find them difficult to remember. Furthermore, some physicians did not see the training program, with its related strategies and tools, as the appropriate measure for improving patient safety; instead, they referred to an increase in the number of healthcare professionals as the best solution. Related to one of the underlying concepts of the team training program, i.e., “the patient as part of the team”, some of the physicians did not understand the meaning of this in practice, which was expressed by one of them as follows:

*“I feel that implicitly in such a concept* [the patient as part of the team], *the patient is just sort of a side scene…and that there is a mistrust towards the healthcare services ingrained in the concept”* (Ph: II, 77).

The physicians felt that the patients were already included in the team and that they were well informed; this was explained to be part of the surgical work culture. They also pointed to the fact that they themselves learned from the patients.

Both RNs, CNAs and physicians experienced the team skill of communication as being vital for the training program. The ISBAR and closed loop tools were experienced as essential for improving communication and providing a common way of conducting communication. At the start of the program, RNs had difficulties remembering the ISBAR acronym and what each of the letters referred to. For the physicians, the ISBAR systematics were more common, which was expressed by one of them as follows:

*“When studying medicine, you learn how to do an admission journal, which is in fact quite similar to ISBAR”* (Ph: II, 21).

The handoff tool was reported to be confusing by RNs and CNAs, which was explained by the fact that the tool was implemented in the last phase of the training program and was therefore not used to its full potential. The debriefing tool was, on the other hand, experienced positively, and the CNAs felt that this tool provided a basis for changes in the ward.

The team skill of situational awareness was something that all the participants felt that they already practiced continuously; however, conceptualizing it through the training program helped them become more conscious of it from their own perspective and from a team perspective. The team skill of mutual support was expressed as being especially important by the RNs. They explained that they had always been supportive of each other but that the content of mutual support in the team training program improved their skills in handling positive and negative situations in the ward.

### The process of implementing the team training program

As a first step of the implementation process of the team training program, all the participating healthcare professionals experienced the one-day training course as an interprofessional initiative that was seen as very useful for the ward. Throughout the different stages of the process, the nursing staff seemed to maintain their motivation for the implementation of the tools and strategies, while the physicians struggled to keep an overview of the different activities and were less actively involved.

Prior to the start of the program, the RNs expressed that by participating, they expected a greater awareness of their own role in teamwork. The physicians felt that they already had good collaboration with the nurses but were humble and acknowledged the fact that there might be issues that they should change to improve. They also expressed a positive attitude towards the simulation-based training.

In the follow-up interviews after six and 12 months, the one-day team training course at the start of the implementation was still mentioned as an excellent initiative by both RNs, CNAs and physicians. Some felt that there was too much information included, yet the overall course program was described by the physicians as being concrete and practically oriented and as laying the foundation for fruitful interprofessional discussions. Furthermore, it was seen as positive that the instructors responsible for the course had clinical practice-based work experience at a hospital. The CNAs felt that the training program covered issues they dealt with on a daily basis without personally reflecting on them; thus, the training course contributed to raising their awareness of their own practices with regards to patient safety:

*“At the team training course, we learned how to repeat messages, for example, when we answer the phone, but this is something that we already do”* (CNA: II, 5).

Everyone felt that it was good to come together, and the team training became something that the participants took part in together across professions. An RN stated as follows:

*“At the team training course, we did not have the attitude that ‘I am the physician doing these tasks, I am the nurse doing these tasks, and I am the CNA so I do these tasks’. It was more a sense of ‘We are in this together, doing it together’"* (RN: III, 25).

The RNs were of the opinion that the fact that all of the professionals had an equal starting point regarding the content of the team training program, with no one possessing more knowledge than anyone else, had an impact on the overall climate of collaboration. According to the physicians, this was the first time that they had gathered across professions for a common course. They felt it useful to be together with the RNs and to get to know each other better:

*“To listen to the nurses and how they perceive the collaboration led to useful reflections on how we do things together, what is good and what can be done differently – that was perhaps the most useful part”* (Ph: II, 38).

The use of simulation-based team training during the one-day course was well received by both RNs and CNAs. The RNs felt that the simulation-based training stimulated better learning, as it improved their ability to carry out clinical tasks. Many of the physicians had already participated in simulation-based trauma training or other courses either in practice or during their education. However, this was the first time that they conducted simulation-based training together with the nursing staff at the ward. Many had been worried about taking part in these simulations, but as a couple of physicians stated:

*“Simulation is a challenge if you are doing it for the first time, but eventually, the level of stress is reduced”* (Ph: II, 16). *“You get emotionally involved quite quickly …and then you are in the situation”* (Ph: II, 14).

Furthermore, in implementing the team training program in the ward, the RNs felt there were many issues to remember. However, with a focus on a single tool each month (the “tool of the month”), it became easier to internalize the tools and use them in their practices. The Weekly Update, the ward’s digital information channel, was used to focus on the TeamSTEPPS tools and strategies. The physicians expressed that they quickly lost the overview of the implementation of the tools; they felt that the program eventually burned out and that this was unavoidable. Their goal was to catch the basic idea behind the tools. To keep the motivation high, part of the implementation process was to include certain tools at the daily interprofessional patient safety whiteboard meetings:

*“At the patient safety whiteboard meetings, we use huddling in relation to the redistribution of tasks if someone feels that they have too much to do while others have available capacity… and we use debriefing if there are challenges”* (RN: III, 58-59).

Furthermore, two two-hour refresher courses were held in the ward, in which RNs and CNAs participated and then reported that such courses were important for maintaining knowledge:

*“The refresher course results in an improved awareness of your actions and how the strategies and tools are labeled in the training program”* (CNA: III, 4*). “For the knowledge from the team training program to be sustained, you sort of need to work it into your practices so that it becomes routine”* (CNA: III, 57).

The physicians held a refresher lesson at a morning meeting, but most of the physicians did not have the opportunity to participate.

### The context in which the team training program is implemented

The team training program was implemented in a surgical ward in which several contextual factors were registered during the adoption and utilization of the related tools and strategies. These factors were facilitating factors, such as a stable workforce, management support and prior quality improvement efforts, and inhibiting factors, such as high workloads and a lack of coherence with other staff groups and wards. Ward information data from Table 1 is used as a supplement when reporting healthcare professionals’ experiences with the contextual factors that influenced the implementation of the team training program.

All the participants felt that prior to the training program, their teamwork was satisfactory and that the different professions involved knew each other well. The ward had work staff stability, and the physicians felt that the RNs were competent and that proper routines existed for training newly employed nurses. The use of humor when implementing the tools was viewed as positive, and both RNs and physicians described a good atmosphere in the ward during the start of the training program. The implementation of the team training program was management supported, and clear initial messages were given to prioritize participation in the program. One physician commented as follows:

*“At the start-up of the team training program, it was sort of a leadership-initiated enthusiasm”* (Ph: II, 23).

The ward had experience with running quality improvement initiatives, and prior to the start-up of the team training program, the ward had implemented patient safety whiteboard meetings and MEWS scoring. The surgical department to which this ward belonged had also started using the Lean methodology a few years ago, with the intention of reducing patient waiting time and improving patient flow and quality. During the team training period, there was a decrease in the number of CNA positions, which was made up for by an increase in the number of RN positions. Ward information data (Table 1) showed an increase in the average bed occupancy rate from 87% before the start of the program to 96% 6 months after the implementation of the team training program. The ward’s busyness was seen as a barrier for the implementation of the program, as commented on by the RNs:

*“It is easy to disregard the tools when it is busy, then you use the things that you feel safe with”* (RN: II, 128-129). *"By using the handoff tool, you at least have to have enough time to think before writing the report, and that is something that we do not always have, you see"* (RN: III, 45).

The RNs were concerned with the time necessary to implement the team training approaches and change their work practices, as these things might be down-prioritized when the ward becomes busy. The physicians also experienced that the summer holidays led to a break in the team training activities and that their overall focus was decreased afterwards. The RNs mentioned the fact that other staff groups, such as ward secretaries, physiotherapists, porters, kitchen and maintenance staff, had not been introduced to the program. The same applied to physicians and healthcare personnel from other wards. They experienced this difference as challenging, i.e., that they as a single ward had received the team training program:

*“We experience that physicians from other wards who have not participated in the program and learned about the communication tools think that we criticize them when we use the closed-loop tool”* (RN: III, 106).

*“When a nurse tries for the tenth time to communicate her concern about the patient's pulse being too high and gets the response from a physician from another ward that this is nothing to stress about and refuses to answer the question… then in the end, there is nothing more to do”* (RN: III, 107).

During the team training period, changes occurred in the composition of the management group. The head surgeon of the urology section left the position, and the chair of the surgical department moved to another position within the hospital and was replaced by the head surgeon of the gastrointestinal surgery section in 50% of cases, thus reducing the amount of clinical work. The clinical nurse specialist took over the role of leader of the change team since the unit nurse manager was allocated to the position of assistant chair of the surgical department. These changes all affected the management focus of the team training program.

### The impact of the team training program on patient safety culture

The team training program reported experiencing impacts on several properties of the ward’s patient safety culture, specifically those related to team performance, open communication, learning from mistakes, team leadership, and patient-centered care. The impacts on patient safety culture was more prominent among RNs and CNAs than among physicians in the ward.

According to the nurses at the ward, the team training program influenced their clinical practices in a useful manner by externalizing their nonarticulated knowledge regarding teamwork in the care culture. The program created a shared understanding and an awareness of how to work together as a team, thereby creating common work practices. RNs experienced an impact on their awareness of how effective communication influences patient safety, including the ability to set expectations across the professional groups involved. They furthermore experienced that the TeamSTEPPS program contributes to a culture of openness, with an increased focus on mutual support across the professions. The physicians expressed a somewhat different view on open communication and team awareness:

*“The team training course opened our eyes about how to communicate with the nursing staff… and what needs to be done to make everyone have a good experience”* (Ph: II, 86-87).

However, another physician said:

*“I do not feel that I got anything in return from the team training course in regard to working in a team, though it might have created an awareness of teamwork among others”* (Ph: II, 64).

The RNs clearly stated that knowledge regarding teamwork skills and the use of teamwork tools had been useful for promoting patient safety culture at the ward:

*“As a nurse, I was relieved when ‘my’ mistake was corrected by the use of the closed-loop tool before the patient got hurt”* (RN: II, 70). “*With a single tool such as the closed-loop, we have identified miscommunication”* (RN: III, 36).

Both RNs and physicians referred to an adverse event that occurred just after the one-day team training course, which concerned an incorrect medication dosage. This event led to a revised procedure based on using tools from the team training program.

The physicians experienced that the RNs used the tools from the program in their communication. A physician referred to a situation in which it was clear that a RN disagreed with him, and he reported that this disagreement was communicated in a manner that had been learned from the team training through the use of the two-challenge rule, which was reported as a positive experience:

*The nurse very clearly voiced her opinion about something that was decided against in the procedure, which I as a physician thought was the right thing to do in this case. I then needed to explain the rationale for doing so. In this case, it was legitimate for the nurse to voice her concern in a clear manner”* (Ph: II, 61).

The CNAs further highlighted the debriefing of the week being conducted at lunch every Friday as a positive contribution to open communication. They reported that it was useful and valuable to talk through the situations they had been through over the course of the week.

Although the patient safety whiteboard meetings were initiated prior to the team training program, RNs and CNAs reported that the program had contributed positively to the completion of the daily interprofessional meetings, in which tools from the program were used. The RNs experienced that they, as team leaders, received feedback from team members more easily and that morning rounds had become more efficient. Initiatives consisting of dedicated nursing staff being responsible for specific patient rooms, headphones for copatients being provided during physicians bedside rounds, rooms being provided for conversations with patients and their caregivers, and declaration forms being filled out by patients prior to their admission to the hospital had been implemented to involve the patients as part of the team. Other patient initiatives were also in the planning:

*“There are plans for initiatives related to the discharge conversation, where the idea is to get the patient more actively engaged in their information needs, and not focusing solely on the standard letter that everyone receives”* (RN: III, 120).

## Discussion

This study has described healthcare professionals’ experiences with the implementation of a longitudinal interprofessional team training program in a surgical ward at a Norwegian hospital. The study is the first to report qualitative results from implementation of the TeamSTEPPS program in Norway and has therefore had a limited scope and sample. Despite this, the in-depth accounts of healthcare professionals’ experiences with implementation should provide valuable knowledge for the practice field internationally and for the design of future implementation studies on a larger scale. The results according to the content of the training program, the process of implementing the program, the context in which the program was implemented, and the impact of the program on patient safety culture indicate a variety of experiences that provide a comprehensive understanding of the implementation as seen by the nurses and physicians who participated in the program.

The findings document that nurses and physicians experienced the ward’s teamwork climate as being good prior to the team training program, which may have been an important factor for the implementation. According to Eddy et al. [23], the organizational context in which teamwork programs are implemented has a significant influence on health professionals’ engagement and learning experiences related to the teamwork efforts. During the implementation period of the current study, the nursing staff appeared to be more engaged in learning and using the new knowledge than were the physicians. While the nursing staff adjusted to the tools and strategies over time and experienced the different components of the program positively, the physicians found it difficult to obtain an overview of these tools and components. However, the physicians appreciated the use of the tools, especially the communication tools, by the nursing staff. This finding may indicate that the physicians considered the content of the team training program to be more important for the nurses than for themselves. This outcome may have to do with the team structure in the surgical ward, where physicians are not situated for extended periods of time, as they might be called to the operating room for surgical procedures, called to conduct consultations, called to documentation tasks, or called to attend to administration duties outside the ward [24].

During the team training program, the nursing staff and physicians were trained together for the first time, and all the healthcare professionals experienced the one-day training course as a successful interprofessional initiative. The reason behind the previous lack of interprofessional training may be the hierarchical structure of healthcare organizations, in which professionalism has traditionally been favored, thereby resulting in professionals working in “silos” [25] and receiving training in professional silos [26]. Having an interprofessional focus is described by Cunningham et al. [4] as the most important contextual factor in a team intervention being effective. The participants of the training program further valued the concrete and practical orientation of the one-day training course, as well as the instructors having a background and experience with clinical practice at a hospital. This outcome is supported by evidence that ensuring the credibility of an intervention highlights that teamwork interventions should be led by health professionals with experience that the participants can relate to, should be comprehensive and relevant to the professional environment, and should be management-supported [4]. According to Thomas, Galla [9], physicians respond best to training conducted by other physicians, in which physicians are engaged as champions who believe in the value of training. Despite the achievement of getting physicians involved in the team training program of the current study, they approached the program with somewhat less enthusiasm than the nursing staff. The changes made to the management group in the surgical ward during the implementation period might have influenced the role of the physicians in the program.

The participants experienced an impact of the team training program on the ward’s patient safety culture in forms of a higher level of awareness about how to work together as a team and openly communicate within and between professional groups. This is also supported by both qualitative [14] and quantitative research results from studying the implementation of the current team training program [27, 28]. Our study results further indicate that the implementation process maintained psychological safety at the ward, which, according to Edmondson [29], is needed to improve patient safety. This may also have supported the healthcare personnel’s effective handling of the adverse event that occurred shortly after the start of the team training. Vifladt et al. [30] found that a positive patient safety culture is also associated with the absence of burnout and the ability to cope with stressful situations.

Some issues regarding study limitations should be noted. Data collection dating back to the period of 2016–2017 may affect the timeliness of the results. However, qualitative research on the implementation of team training interventions is mainly missing and we therefore see the study results as a relevant contribution to the evidence base. The limited number of participants and the variation in the focus group sizes may have influenced the results. However, the completion of ten focus group interviews at three time intervals over 1 year combined with contextual ward information provided us with rich data material. Nevertheless, conducting additional partial participant observations could further have strengthened our analysis. The application of a qualitative design restricts the generalization of our study results. However, the results provide an in-depth understanding of health professionals’ experiences with the implementation of a longitudinal team training program that may also offer relevant knowledge for other similar healthcare settings.

## Conclusions

According to the experiences of nurses and physicians, the implementation of a team training program in a surgical hospital ward can be described as a comprehensive activity related to the content of the training program, the process of implementation, the context in which implementation takes place, and the impact of the program. The *content* of the team training program was experienced as relevant yet complex, more so by nurses than physicians. Furthermore, time and effort were needed for the professionals involved to understand the full range of tools and strategies in the program. The interprofessional focus of the implementation *process* was experiences as positive, although the nursing staff maintained their motivation throughout the program period better than the physicians did. The *context* in which the team training program was implemented was characterized by workforce stability and high workloads, the first contributing positively to implementation while the latter caused challenges related to the time and effort needed for successful implementation. Finally, implementation of the team training program was experienced as having an *impact* on the patient safety culture at the surgical ward through increasing healthcare professionals’ awareness of teamwork and contributing with more open communication among them.

The results highlight how and why a team training program may work supporting the transferability of valuable knowledge to clinical practice in planning team training and supporting sustainability of the training. In supporting sustainability of the team training program we suggest the following actions: Simplification of the team training content to make it easier comprehensible, repetition of team training activities to stimulate motivation throughout the implementation process, and to strive towards workforce stability to secure optimal contextual conditions for the team training program. Furthermore, leadership support and responsibility are vital for the sustainability of the program. To further support transferability and sustainability of the team training program more research is needed.

## Supplementary Information

**Additional file 1.**

## Data Availability

The datasets generated and analysed during the current study are not publicly available due to the consistency of a large number of the qualitative interview transcripts being in Norwegian but are available from the corresponding author on reasonable request.

## Abbreviations

TeamSTEPPS: Team Strategies and Tools to Enhance Performance and Patient Safety
RN: Registered nurse
CNA: Certified nurse assistant
