# Supplementary material for: Longitudinal team training program in a Norwegian surgical ward: a qualitative study of nurses’ and physicians’ experiences with implementation
Source: BMC Health Serv Res. 2021 Jul 23;21:725. doi: 10.1186/s12913-021-06732-6 (PMC8299676; doi:10.1186/s12913-021-06732-6)
Supplement: Supplementary file 1 — Additional file 1. [file 12913_2021_6732_MOESM1_ESM.docx]

**Additional file 1**

**Interview guide**

**Prior to the intervention**

- What are your opinions on participating in the team training program?
- How do you think a team training program can promote quality and patient safety at the ward?

**Six months after the intervention**

- How do you experience participating in the team training program to improve patient safety culture at the ward?
- How did you experience the one-day team training course at the university campus?
- How do you experience the implementation of the program at the ward?
- How do you experience that participating in the team training program can contribute to promote patient safety at the ward?

**Twelve months after the intervention**

- How do you experience participating in the team training program to improve patient safety culture at the ward?
- How do you experience the implementation of the program at the ward?
- How did you experience the TeamSTEPPS^®^ refresher courses?
- How do you experience that participating in the team training program can contributes to promote patient safety at the ward?
